# Age and Sex-Related Changes in Human First-Trimester Placenta Transcriptome and Insights into Adaptative Responses to Increased Oxygen

**DOI:** 10.3390/ijms22062901

**Published:** 2021-03-12

**Authors:** Fulin Liu, Christelle Simasotchi, Françoise Vibert, Wencan Zhu, Sophie Gil, Séverine A. Degrelle, Thierry Fournier

**Affiliations:** 1 Pathophysiology & Pharmacotoxicology of the Human Placenta, Pre & Postnatal Microbiota, Université de Paris, INSERM, 3PHM, F-75006 Paris, France; fulin.liu@etu.u-paris.fr (F.L.); christelle.simasotchi@parisdescartes.fr (C.S.); francoise.vibert@parisdescartes.fr (F.V.); sophie.gil@parisdescartes.fr (S.G.); severine.degrelle@inserm.fr (S.A.D.); 2 Fondation PremUp, F-75006 Paris, France; 3 UMR Applied Mathematics & Informatics, AgroParisTech-Université Paris-Saclay, F-75005 Paris, France; wencan.zhu@agroparistech.fr; 4 Inovarion, F-75005 Paris, France

**Keywords:** placenta, trophoblast, oxygen, RNAseq, WGCNA, DESeq2

## Abstract

Physiological oxygen tension rises dramatically in the placenta between 8 and 14 weeks of gestation. Abnormalities in this period can lead to gestational diseases, whose underlying mechanisms remain unclear. We explored the changes at mRNA level by comparing the transcriptomes of human placentas at 8–10 gestational weeks and 12–14 gestational weeks. A total of 20 samples were collected and divided equally into four groups based on sex and age. Cytotrophoblasts were isolated and sequenced using RNAseq. Key genes were identified using two different methods: DESeq2 and weighted gene co-expression network analysis (WGCNA). We also constructed a local database of known targets of hypoxia-inducible factor (HIF) subunits, alpha and beta, to investigate expression patterns likely linked with changes in oxygen. Patterns of gene enrichment in and among the four groups were analyzed based on annotations of gene ontology (GO) and KEGG pathways. We characterized the similarities and differences between the enrichment patterns revealed by the two methods and the two conditions (age and sex), as well as those associated with HIF targets. Our results provide a broad perspective of the processes that are active in cytotrophoblasts during the rise in physiological oxygen, which should benefit efforts to discover possible drug-targeted genes or pathways in the human placenta.

## 1. Introduction

The human placenta plays a pivotal role in development by regulating the exchange of nutrients, gas, and waste between the mother and the fetus. Normal development of the placenta can be divided into three trimesters, of which the first lays the foundation for all subsequent processes. Indeed, at the very beginning of the first trimester, it is the yolk sac that first establishes the supply of oxygen and nutrients to the embryo, and this role is gradually taken over by the growing placenta. From day 13 post-coitum, the placental villus begins to form and branch, and is subsequently infiltrated by allantoic blood vessels [1]. This process involves interactions between fetal and maternal tissues and specifically involves the trophectoderm stem cells, which then evolve into several different trophoblast cell lineages that constitute the main part of the placenta [2,3]. The evolution of this process is complex and intricately regulated by O_2_ tension, especially in the period from 8 to 12 gestational weeks (GW) [4]. Defects in this process can lead to diseases of pregnancy such as spontaneous abortion, preterm birth, and preeclampsia [5,6].

The oxygen tension in the intervillous space rises dramatically from 2 to 3% around 8–10 GW to more than 6% after 12 weeks [4]. Oxygen, and the oxidative stress that accompanies it, plays an important role in the positive or negative development and growth of chorionic villi. Histopathological research has shown that villous cytotrophoblast thrives under the intrauterine hypoxia of the first trimester [7]. Instead, the hyperoxic state in the placenta inhibits capillary branching, the formation of sinusoids, and the differentiation of villous cytotrophoblast [8]. The increase in oxygen tension at 8–12 GW is driven by remodeling of the maternal spiral arteries by invasive extravillous trophoblast. The supply of blood flow allows a rapid increase in O_2_ tension in the intervillous space [2,3], thus satisfying the high oxygen demands of the placenta, which represents at least 30% of the total amount of the utero-placental unit [9,10].

Oxygen is consumed in the mitochondria, and recent research has uncovered sexually dimorphic aspects of this process, particularly in cases of placental mitochondrial dysfunction [11,12]. For example, male guinea pig placenta is more susceptible to respiratory complex chain disruption under gestational hypoxia [13]. It is well recognized that the placenta acts similar to an orchestrator, adapting morphologically and hormonally in response to variations in the environment [14]. Hypoxia (2–3% O_2_), as an ever-present challenge throughout the duration of pregnancy, functions as a two-edged sword for the development of the placenta. While suitable levels of hypoxia/low O_2_ tension induce the remodeling of maternal spiral arteries, extreme hypoxia disables the remodeling and can result in adverse conditions such as preeclampsia [5,6] and insufficient hypoxia leads to over-consumption of the nutrients delivered into the intervillous space for the nourishment of the fetus [15]. Taken together, these findings indicate the existence of different strategies for male and female placentas in coping with variable oxygen conditions.

There are three conventional oxygen-sensitive pathways that regulate gene expression in the placenta under low O_2_ tension: hypoxia inducible factors (HIFs), unfolded protein response (UPR), and mammalian target of rapamycin (mTOR). Their activation regulates gene expression, metabolic homeostasis, and cell survival [16]. Of these, the most studied signaling pathway is HIF, which is a heterodimeric transcription factor composed of two subunits, HIF-α and HIF-β (alias: ARNT, aryl hydrocarbon receptor nuclear translocator) [17]. HIF-α and HIF-β bind to specific promoter sequence elements to activate target gene transcription and thus enable many different cellular processes to respond to hypoxia during early placentation [18,19]. Interestingly, the response to hypoxia has been shown to differ between male and female placentas, particularly with respect to energy metabolism and angiogenesis. The female placenta was described to activate more protective mechanisms to increase the availability of nutrients for fetal metabolic development [11], whereas in preeclamptic pregnancies, male placentas showed stronger reductions in pro-angiogenic markers than female placentas did [20]. However, such patterns of sexual dimorphism are still poorly documented, and at a fundamental level, the steep increase in oxygen tension in the human placenta remains only incompletely understood.

Here, we applied next-generation sequencing technology to sequence human villous cytotrophoblasts freshly isolated from placentas at either 8–10 GW or 12–14 GW with the goal of assessing changes in gene expression and the influence of placental sex differences in this key physiological period.

## 2. Results

### 2.1. Identification of Outliers in Samples

**Figure 1:**
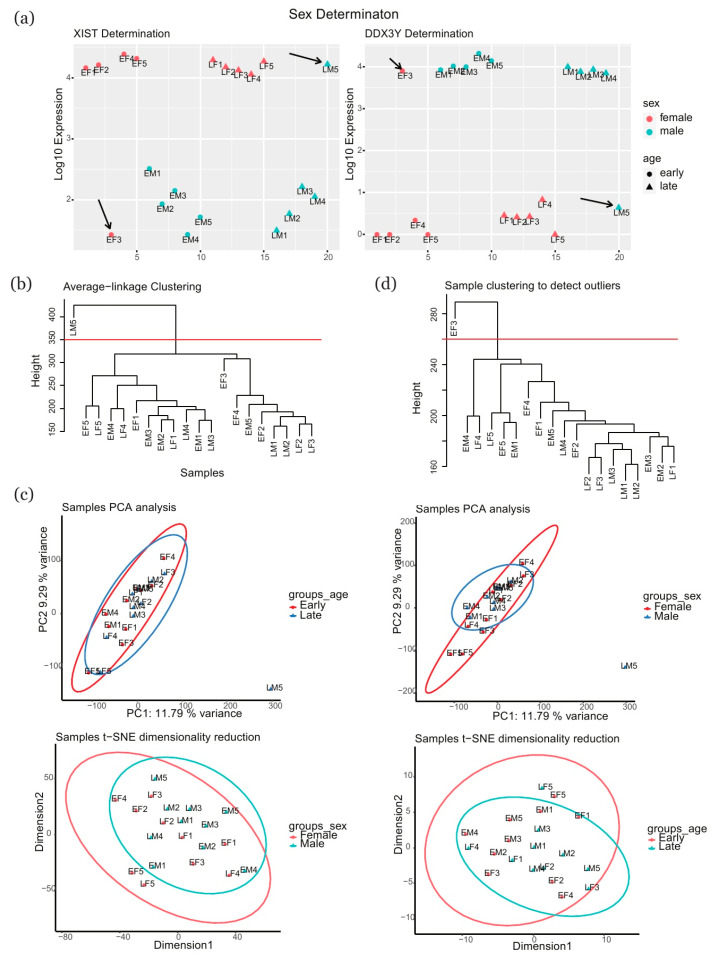
Sex determination and sample clustering. (**a**) Expression of the sex-linked genes *XIST* and *DDX3Y* in samples. (**b**) Optimal number of clusters for samples and sample clustering according to K-means clustering algorithm. (**c**) PCA analysis and t-SNE dimensionality reduction in samples. (**d**) Sample re-clustering using WGCNA method without LM5. PCA: principal components analysis; t-SNE: t-distributed stochastic neighbor embedding; EF: early female; EM: early male; LF: late female; LM: late male; WGCNA: weighted gene co-expression network analysis.

Our RNAseq dataset has been deposited in the Gene Expression Omnibus public repository (https://www.ncbi.nlm.nih.gov/geo/) under the accession number GSE163023 (https://www.ncbi.nlm.nih.gov/geo/query/acc.cgi?acc=GSE163023). The quality report generated by FastQC indicated that the raw data were appropriate for further use: the majority of plots were well above phred score 30 (green region), the average quality was in single peak, a well-overlapped single hump was shown in the center, and the data remained consistent before and after normalization (Supplementary Figure S1). First, we retrieved the expression of *XIST* and *DDX3Y* in each sample and used this information to validate the sex designations obtained with PCR. The results of the two methods were in agreement for the vast majority of samples, with the exception of samples “LM5” and “EF3” (Figure 1a). Next, to evaluate the quality of the samples and detect outliers, we clustered the samples for classification. The number of sample clusters was generally suggested to be two according to random k-means clustering, while the use of distance clustering identified sample “LM5” as an outlier (Figure 1b). “LM5” was also classified as an outlier according to separate PCA analyses of age and sex, while no difference was indicated by the t-SNE analysis (Figure 1c). “EF3” was identified as an outlier in a subsequent analysis of distance clustering in weighted gene co-expression network analysis (WGCNA) (Figure 1d). Therefore, “LM5” and “EF3” were excluded from the dataset, and the remaining samples were subjected to further gene expression analyses.

### 2.2. Identification of Key Genes and Term Enrichment Using DESeq2

**Figure 2:**
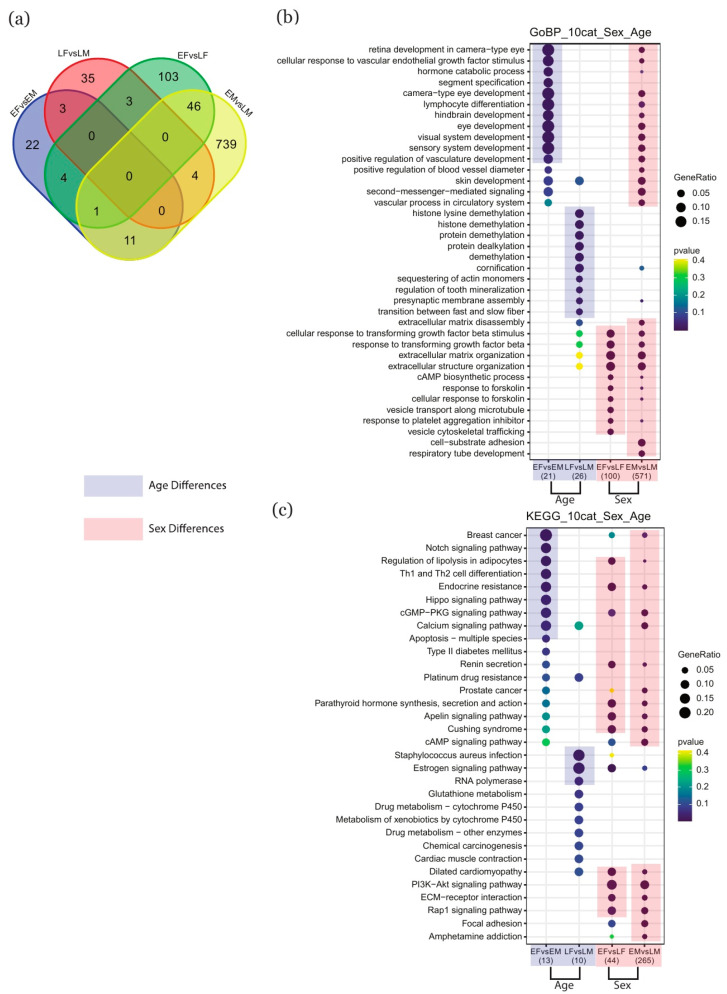
Identification of differentially expressed genes (DEGs) based on the DESeq2 method and assessment of term enrichment. (**a**) Venn diagram showing DEGs from different comparisons. (**b**,**c**) Enrichment and comparison of GO terms and KEGG pathways, respectively, based on DEGs. The size of a point indicates the magnitude of enrichment; plum shading represents an association with age, and coral shading represents an association with sex. “E” stands for “Early”, “L” for “Late”, “F” for “Female”, and “M” for “Male”. GO: gene ontology; KEGG: Kyoto encyclopedia of genes and genomes.

The DESeq2 method, based on the DESeq2 R package, was applied to detect key genes that were differentially expressed between different groups. This analysis identified 15 key genes with expression differences in the comparison of *Female* versus *Male*, 457 key genes for *Early* versus *Late*, 45 key genes for *Late Female (LF)* versus *Late Male (LM)*, 41 key genes for *Early Female (EF)* versus *Early Male (EM)*, 157 key genes for *EF* versus *LF*, and 801 key genes for *EM* versus *LM*. The expression patterns were then used to construct heatmaps (Supplementary Figure S2a–f). The Venn diagram in Figure 2a depicts the intersection of key genes among comparisons. These key genes were then submitted to analyses of enrichment and term comparison, specifically with respect to GO biological processes and KEGG pathways (Figure 2b,c). In addition, we also analyzed enrichment in GO cellular components and GO molecular functions, and these comparisons are detailed in the supplementary materials (Supplementary Figure S3). 

For GO biological processes, the early-stage placentas demonstrated enrichment in aspects of organic development such as “camera-type eye development”, “lymphocyte differentiation”, and “hindbrain development”, while later-stage tissues were mainly enriched in processes related to biological regulation, such as “histone demethylation”, “protein demethylation”, and “protein dealkylation”. When we instead compared the tissues based on sex, we found that the male and female placentas shared many of the same terms for cellular processes and biological regulation, such as “response to transforming growth factor beta”, “extracellular matrix organization”, and “response to forskolin”. We did, however, detect additional enrichment in males in certain processes related to organic development, such as “eye development”, “lymphocyte differentiation”, and “hindbrain development” (Figure 2b).

For KEGG pathways, when we only examined the effect of age, we observed that early-stage samples were characterized by enrichment in regulatory pathways, such as the “notch signaling pathway”, “hippo signaling pathway”, and “calcium signaling pathways”, while in late-stage samples, enrichment was noted in pathways associated with “staphylococcus aureus infection”, “estrogen signaling pathway”, and “RNA polymerase”. Instead, when we compared the male and female samples, we found that the sexes were very similar in their biological regulation; both sexes demonstrated enrichment in terms such as “regulation of lipolysis in adipocytes”, “endocrine resistance”, “cGMP-PKG signaling pathway”, “apelin signaling pathway”, and “PI3K-Akt signaling pathway” (Figure 2c).

### 2.3. Selection of Key Genes Based on WGCNA Method

**Figure 3:**
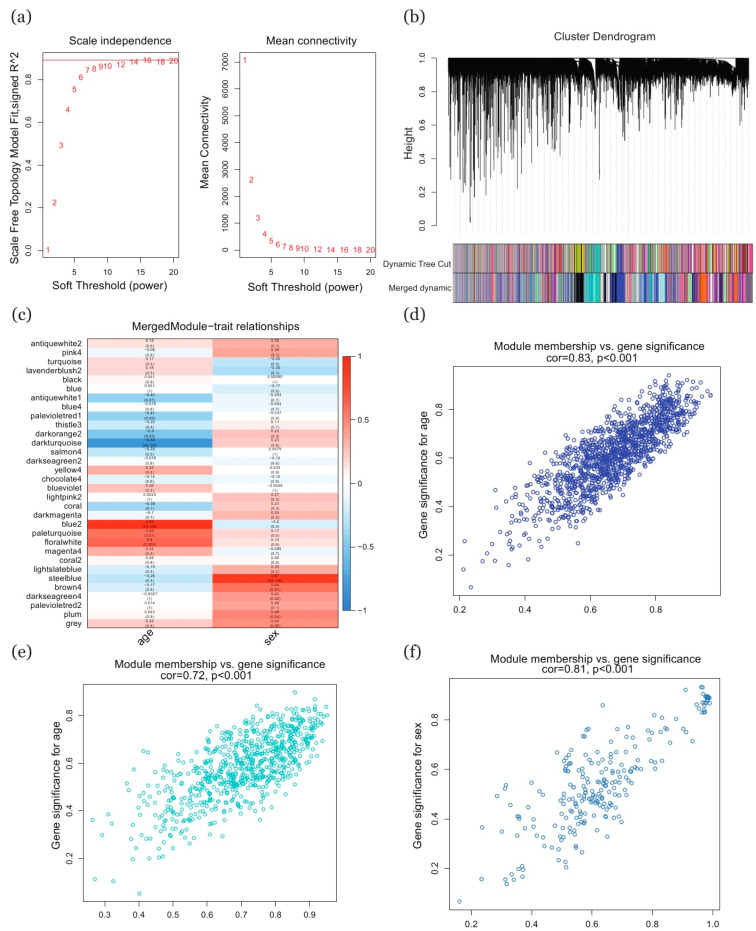
Selection of key genes using the WGCNA method. (**a**) Soft-thresholding power selection from a range of power values from 1 to 20. (**b**) Cluster dendrogram of genes obtained through dissimilarity clustering based on consensus topological overlap. The first row below the dendrogram represents the unmerged colored modules while the second depicts the merged colored modules. (**c**) Heatmap of correlation coefficients of the association between each module and each trait, with rows representing the modules and columns representing the traits. (**d**,**e**) Scatterplots for merged modules (blue, dark turquoise) associated with age. (**f**) Scatterplot for merged module (steel blue) associated with sex. The criteria for selecting merged modules were a correlation coefficient higher than 0.6 and a *p* value less than 0.05. WGCNA: weighted gene co-expression network analysis.

We also used the WGCNA method to detect key genes that demonstrated differences in expression between groups. The optimal soft thresholding power value was set as 16, which was the value at which measurements of scale independence exceeded the required threshold of 0.9 (Figure 3a). The original clustered modules, which each represented a set of eigengenes, and the merged modules are presented in a cluster dendrogram (Figure 3b). The correlation coefficients between the merged modules and the traits under consideration (age, sex) are shown in the form of a heatmap, in which red denotes a positive relationship and blue denotes a negative one (Figure 3c). Following our selection criteria (correlation coefficient higher than 0.6 and *p* value less than 0.05), the “blue” and “dark turquoise” modules were retained for their association with age (Figure 3d,e), while the “steel blue” module was linked with sex (Figure 3f). Within the two modules associated with age, there were 2015 key genes, while the sex-related module contained 233. Supplementary Figure S4 contains a heatmap depicting the expression of genes in the modules for each of the traits.

### 2.4. Comparison of Enriched Terms between DESeq2 and WGCNA

**Figure 4:**
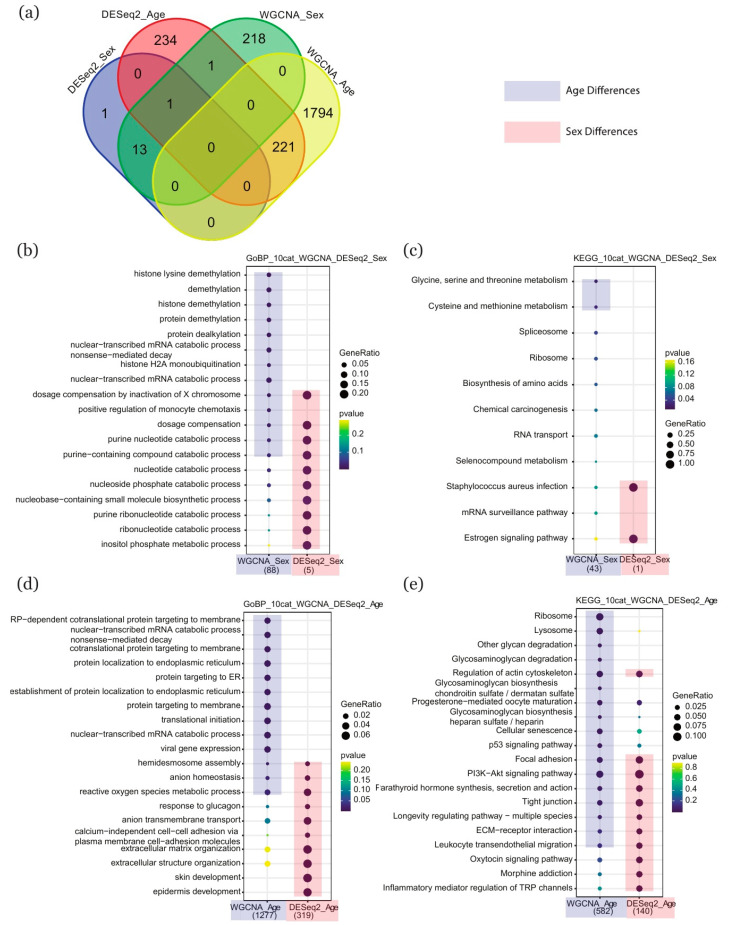
Comparison of enriched GO terms and KEGG pathways in separate sets of genes selected by DESeq2 and WGCNA. (**a**) Venn diagram showing overlap between the two methods in the key genes associated with age- and sex-related differences. (**b**,**c**) Sex-associated enrichment in GO terms and KEGG pathways identified by the two methods. (**d**,**e**) Age-associated enrichment in GO terms and KEGG pathways identified by the two methods. The size of each point represents the magnitude of enrichment, plum shading represents an association with age, and coral shading represents an association with sex. GO: gene ontology; KEGG: Kyoto encyclopedia of genes and genomes; WGCNA: weighted gene co-expression network analysis.

To compare the two methods, we extracted the key genes from each (DESeq2_Sex: 15, DESeq2_Age: 457; WGCNA_Sex: 233, WGCNA_Age: 2015) and compared the patterns of enrichment in GO biological processes and KEGG pathways. The Venn diagram in Figure 4a shows the intersection among groups from different methods and conditions. Using the key sex-related (Figure 4b,c) or age-related (Figure 4d,e) genes highlighted by the two methods, we compared the resulting patterns of enrichment in GO biological processes and KEGG pathways revealed by each approach. We performed a similar analysis of enrichment with respect to GO cellular components and GO molecular functions, which is included in the supplementary materials (Supplementary Figure S5).

For GO biological processes, both methods revealed a relationship between sex and enrichment in “dosage compensation by inactivation of X chromosome” and “purine nucleotide catabolic process”. Instead, the WGCNA_Sex dataset highlighted enrichment in regulatory processes such as “histone lysine demethylation”, “demethylation”, and “histone demethylation”, and the DESeq2_Sex dataset was mainly characterized by metabolic processes such as “nucleotide catabolic process”, “nucleotide phosphate catabolic process”, and “ribonucleotide catabolic process” (Figure 4b). With respect to age, both methods revealed enrichment in “hemidesmosome assembly”, “anion homeostasis”, and “reactive oxygen species metabolic process”, while the WGCNA_Age dataset was mainly characterized by cellular processes such as “protein localization to endoplasmic reticulum”, “protein targeting to membrane”, and “RP-dependent cotranslational protein targeting to membrane” and the DESeq2_Age dataset highlighted cellular processes such as “response to glucagon”, “anion transmembrane transport”, and “extracellular matrix organization” (Figure 4d).

For KEGG pathways, there was no overlap between the WGCNA and DESeq2 datasets for sex-related genes. The analysis of enrichment in the WGCNA_Sex dataset revealed the importance of metabolic processes such as “glycine, serine, and threonine metabolism” and “cysteine and methionine metabolism”, while the DESeq2_Sex dataset was characterized by biological processes such as “staphylococcus aureus infection” and the “estrogen signaling pathway” (Figure 4c). With respect to age-related genes, both methods highlighted enrichment in “Focal adhesion”, “PI3K-Akt signaling pathway”, and “Tight junction”, while the WGCNA_Age dataset was mainly characterized by metabolic processes such as “glycan degradation”, “glycosaminoglycan biosynthesis”, and “lysosome” and the DESeq2_Age dataset featured regulatory pathways such as “oxytocin signaling pathway”, “morphine addiction”, and “inflammatory mediator regulation of TRP channels” (Figure 4e).

### 2.5. Enrichment in HIF Targets

**Figure 5:**
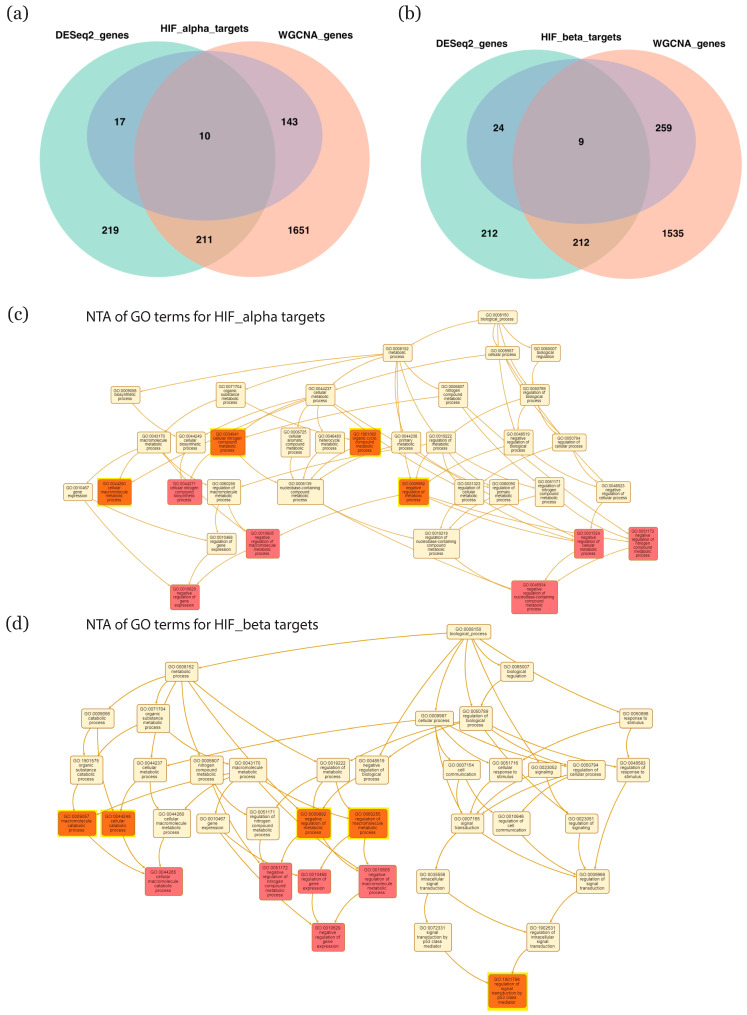

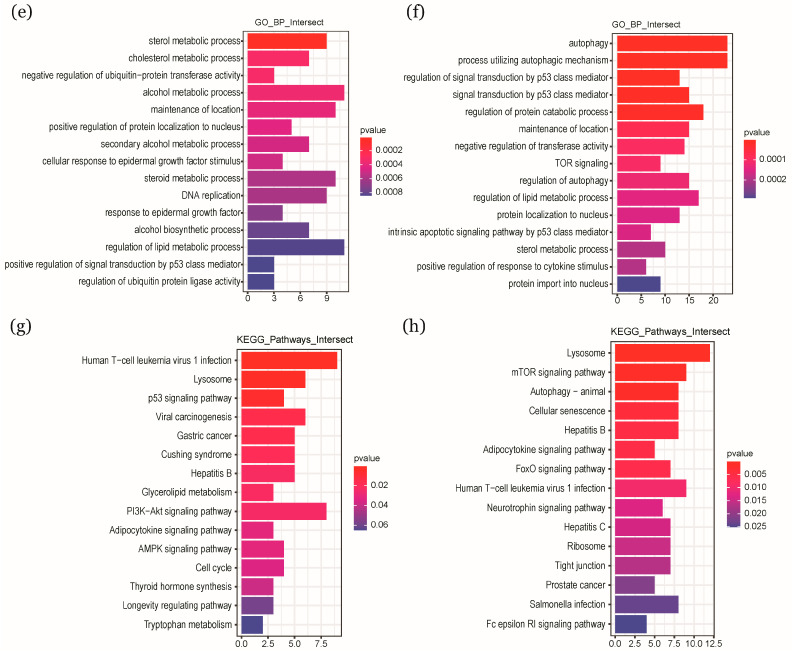
Enrichment patterns in HIF targets. (**a**,**b**) Venn diagram showing intersections between key genes selected by DESeq2 or WGCNA and targets of HIF-1α or HIF-1β, respectively. (**c**,**d**) Network topology analysis for enriched GO biological processes associated with targets of HIF-1α or HIF-1β, respectively. (**e**,**g**) Enriched GO terms and KEGG pathways, respectively, associated with targets of HIF-1α (**f**,**h**) Enriched GO terms and KEGG pathways, respectively, associated with targets of HIF-1β. Significance was defined as P value less than 0.05. HIF: hypoxia-inducible factor; GO: gene ontology; KEGG: Kyoto encyclopedia of genes and genomes; WGCNA: weighted gene co-expression network analysis.

As all samples were collected during a period in which cytotrophoblasts are adapting to an increase in O_2_ levels, we specifically investigated the involvement of HIF in the biological processes in these cells. HIF transcriptional activity depends on its alpha and beta subunits; we therefore assembled a database of all published or predicted targets of HIF-1α or HIF-1β from the literature and public databases (details in Supplementary Table S1). For HIF-1α, we retrieved a total of 2213 targets, of which 170 corresponded to key genes identified by our analyses (Figure 5a). For HIF-1β, we retrieved a total of 3134 targets, of which 294 corresponded to key genes (Figure 5b). Of these 464 key genes, 461 were age-associated and three were sex-associated. Using the groups of HIF-1α or HIF-1β targets, we next performed network topology analyses of the GO biological processes associated with each group. For targets of HIF-1α, the resulting hierarchy of GO biological processes highlighted, in order, the terms “cellular macromolecule metabolic process”, “cellular nitrogen compound metabolic process”, “organic cyclic compound metabolic process”, and “negative regulation of metabolic process” (Figure 5c). For targets of HIF-1β, instead, the dominant hierarchy of terms featured “regulation of signal transduction of p53 class mediator”, “regulation of macromolecule metabolic process”, “negative regulation of metabolic process”, “cellular catabolic process” and “macromolecule catabolic process” (Figure 5d).

Using clusterProfiler, we also analyzed enrichment in GO biological processes and KEGG pathways within each group of targets. Figure 5e,g depict enrichment patterns for the alpha subunit, while Figure 5f,h provide the same information for the beta subunit. With respect to GO biological processes, targets of HIF-1α demonstrated enrichment in terms that were mainly oriented around metabolic processes, such as “sterol metabolic process”, “cholesterol metabolic process”, and “alcohol metabolic process” (Figure 5e). Instead, targets of HIF-1β were mostly associated with terms linked with biological regulation such as “autophagy”, “regulation of signal transduction by p53 class mediator”, and “regulation of protein catabolic process” (Figure 5f). With respect to KEGG pathways, targets of the alpha subunit were associated with enrichment in regulatory pathways such as “human T-cell leukemia virus 1 infection”, “lysosome”, and “p53 signaling pathway” (Figure 5g), while targets of the beta subunit were linked with pathways such as “lysosome”, “mTOR signaling pathway”, and “autophagy” (Figure 5h).

### 2.6. Selection of GO Terms and Associated Genes Most Affected by HIF

**Figure 6:**
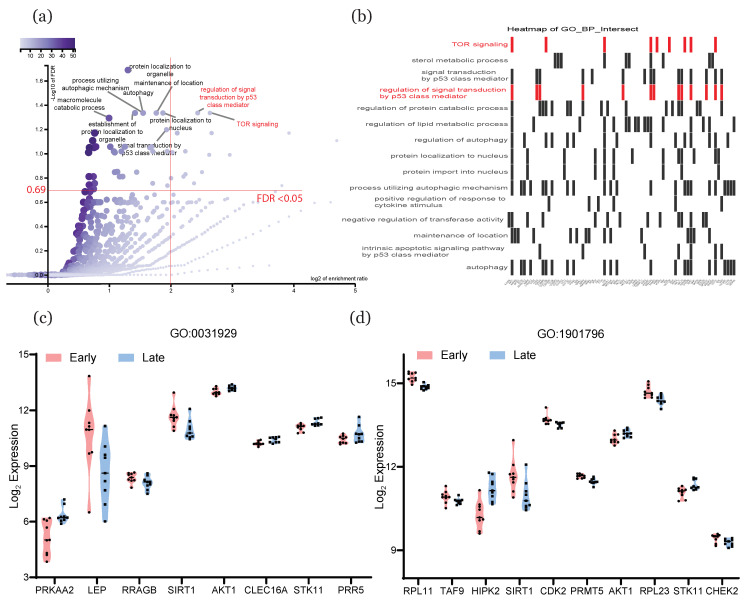
Selection of GO terms and associated genes that were most affected by HIF. (**a**) Scatterplot of GO terms, with the y-axis representing −log10 FDR and the x-axis representing log2 enrichment ratio. (**b**) Heatmap of enriched GO terms among HIF targets, with GO terms on the y-axis and the genes associated with each term on the x-axis. (**c**,**d**) Expression patterns in early and late placenta samples of genes linked to “regulation of signal transduction by p53 class mediator” (GO:0031929) and “TOR signaling” (GO:1901796), respectively. GO: gene ontology; HIF: hypoxia-inducible factors; FDR: false discovery rate.

To identify the pathways and genes that were most affected by HIF, we restricted our set of enriched terms by applying more stringent selection criteria: an FDR threshold less than 0.05 (−log10 of FDR more than 0.69) and log2 enrichment ratio higher than 2. Of all the GO terms and KEGG pathways that were linked with HIF targets, the only ones that met these criteria were two GO terms associated with HIF-1β: “regulation of signal transduction by p53 class mediator” and “TOR signaling” (Figure 6a, in the top-right quadrant). Additional information regarding further evaluation of GO terms and KEGG pathways can be found in the supplementary materials (Supplementary Figure S6). We then visualized the associations between these pathways and their target genes in a heatmap, with the y-axis representing the pathways and the x-axis representing the genes involved (in red, Figure 6b). Expression data were extracted for the genes associated with these terms, and the changes with respect to age were plotted in violin graphs (Figure 6c,d).

## 3. Discussion

The human placenta functions as a biological barrier, between the mother and the fetus, that facilitates the exchange of gases, nutrients, and wastes. Throughout pregnancy, perfusion of the chorionic villi by blood from the uterine spiral arteries is essential for placental development and, therefore, for the exchanges between maternal and fetal blood. Physiological obturation of the uterine spiral arteries by extravillous trophoblasts plugs in the early first trimester is critical for the growth of chorionic villi and development of feto-placental vasculatures. Moreover, the low physiological O_2_ tension within the intervillous space before 10 GW limits the oxidative stress of the chorionic villi, which at this point do not express enzymes that protect from reactive oxygen species (ROS). From 10 to 12 GW, the trophoblastic plugs disintegrate, allowing oxygenated maternal blood into the intervillous space and dramatically raising the oxygen tension. Proper blood flow is thus shaped by oxygen conditions in early placentation, especially in the period from 8–12 GW when oxygen levels increase dramatically [4]. Oxygen levels also regulate the invasion of extravillous cytotrophoblasts into the maternal uterus [21,22]. Abnormalities in these early physiological processes can lead to pregnancy diseases such as spontaneous abortion, preterm birth, intrauterine growth restriction and preeclampsia [5,6]. In our study, we focused on the development of normal villous cytotrophoblasts from 8 to 14 GW, using next generation sequencing technology to investigate changes in gene expression between the early (8–10 GW) and late (12–14 GW) stage of this period.

Although several sequencing datasets have been published for cytotrophoblasts in this period, they have all been focused on different hypotheses. Our data provide what is to our knowledge the first overview of the changes in gene expression that accompany the dramatic increase in oxygen levels from 8 to 14 GW. A search of publicly available GEO DataSets (https://www.ncbi.nlm.nih.gov/gds/) with the key words “human AND placenta” returned a total of 320 accessions that contained microarray or RNAseq data from *Homo sapiens*. Of these, however, only a few had performed tissue-specific sequencing or shared even superficial similarities with our study [23,24,25,26,27]. For example, Sitras et al. [25] compared microarray-based transcriptomes of first trimester and term human placentas, but their analysis focused only on gestational age and did not investigate the effects of sex. Soncin et al. [26] performed genome-wide expression profiling of human placentas from 4 to 16 GW and at 39 GW with the goal of performing a comparative analysis of mouse and human placentas across gestation, but they did not specifically investigate oxygen-related mechanisms or sexual dimorphism. Of the studies that have examined the effects of hypoxia in human placentas, the samples that were sequenced—term trophoblasts [28], first trimester trophoblasts [29], and extravillous trophoblast cultured at different concentrations of oxygen [30]—all differed from the present study.

To investigate sexual dimorphism in the gene expression of human placentas, Braun et al. [23] surveyed the human chorionic villus transcriptome from 11 to 16 GW for sex-linked signatures, with the goal of characterizing genes that are differentially expressed within the first window of increasing testis-derived androgen production in the male fetus. That study was similar to two others [24,27] that also focused on sex-based differences in the human placental transcriptome in the late first trimester, with minor differences in the cells or tissues examined. Here, we considered not only differences based on sex but also those based on age in our evaluation of the effect of oxygen tension. In sum, although previous studies have examined the same period of development as the present study, the purposes of their investigations differed substantially. Furthermore, no previous study has taken into consideration the limitations of the use of only one method in exploring these kinds of data. With respect to this kind of analysis, the Limma, edgeR or DESeq2 methods represent improvements over Student’s test, but ignore the data connections in the matrix that arise as a result of topology [31]. To address these limitations, our analysis followed the example of several previous studies [32,33,34,35] and combined the methods of DESeq2 and WGCNA.

With regard to gestational age, the results of our analysis of term enrichment were largely consistent with reports from the literature. For example, our results indicated the involvement of the “PI3K-Akt signaling pathway”, which has been implicated in the decidualization of trophoblasts in early pregnancy [36]; the “hippo signaling pathway”, which has been reported to control the self-renewal of cytotrophoblasts and protect against early pregnancy loss in humans [37]; the “cAMP signaling pathway” and “rap1 signaling pathway”, which regulate placental cell fusion [38,39]; the “notch signaling pathway”, which plays a critical role in the motility and differentiation of cytotrophoblasts [40]. Our analyses also highlighted the terms “estrogen signaling pathway” and “protein targeting to ER”, which may reflect reports that increased estrogen levels have a major role in regulating placental secretion of macrophage migration inhibitory factor, a proinflammatory cytokine involved in pregnancy [41]. Finally, we compared our data to those of Soncin et al. [26] by extracting the corresponding samples (8–10 GW and 12–14 GW) from GSE100051 and performing the DESeq2 analysis. This highlighted a total of 185 key genes (Supplementary Figure S7b), representing enrichment in 145 terms of GO biological processes, compared to 387 terms in our study enriched through 457 key genes in the same method (Supplementary Figure S7c,d). Our results suggested more abundant terms in each class of GO biological processes, which revealed much more important biological processes. This discrepancy could be an indicator of a higher degree of resolution and accuracy in our study than in this previous work.

From the DESeq2 analysis that considered only differences related to sex, and not age, we obtained only 15 key genes. What was interesting, though, was that this number apparently increased after we divided the groups based on sex and compared the early and late stages separately (41 genes in early and 44 genes in late). That is, the difference between sexes was partly obscured in the age-mixed set of samples, which indicated that, in the first trimester of pregnancy, gestational age exerted a stronger influence on the development of the placenta than sexual dimorphism did. This was similar to a report that age appeared to be more dominant than sex in affecting early fetal lung developments from 54–127 days post-conception [42]. When we examined these 85 (41 + 44) key genes, we found that many of them related directly to the sex chromosome (either X-linked or Y-linked). Within this set of genes, we detected enrichment in GO terms that were mainly associated with catabolic processes, as well as the KEGG pathway “estrogen signaling pathway”, which was similar to previously published results [24]. When this approach was combined with WGCNA, the scope of enriched activities was extended to post-transcriptional modifications, such as “ubiquitination”, “demethylation”, and “dealkylation”, which was also consistent with previous research [43,44,45,46].

In the placenta, oxygen-sensitive pathways are regulated by the actions of HIF on downstream genes. We thus specifically examined the key genes highlighted by our analyses to identify potential HIF targets. Of the 248 key genes linked with sex-based differences identified by DESeq2 and WGCNA, there were only three HIF targets in the intersections: FA complementation group C (FANCC), asparaginyl-tRNA synthetase 2 (NARS2), and RAB38; the latter two genes are known to be active in mitochondria, which could explain their potential correlations with oxygen metabolism. Overall, though, the small number of sexually dimorphic HIF targets could suggest that there is little difference in hypoxia-related biological processes between early male and female placentation. We thus excluded the sex-related genes from our enrichment analyses and focused only on HIF targets that demonstrated age-related expression changes. For the targets of HIF-1α, enrichment analyses highlighted GO terms that were predominantly associated with metabolic processes, and to a lesser extent with biological regulation, while targets of HIF-1β demonstrated the opposite pattern. This could indicate the existence of complementary roles for HIF-1α and HIF-1β under hypoxic conditions [47]. With respect to enriched KEGG pathways (e.g., “p53 signaling pathway”, “PI3K-Akt pathway”, “mTOR signaling pathway”, and “autophagy), our results were largely in accordance with previous studies. Inhibition of the mTOR signaling pathway has been linked to hypoxia-induced cellular energy conservation, i.e., a decrease in ATP consumption when oxygen is limited [16]. Autophagy plays a critical role in maintaining homeostasis by balancing HIF1α-mediated cellular energy consumption [48], and downregulation of the p53 signaling pathway was reported to drive autophagy in the syncytiotrophoblast [49]. Inhibition of the PI3K-Akt pathway predisposed first-trimester trophoblasts to oxygen-induced cell death [50]. From this set of terms, we wanted to select the most critical for further exploration; to do this, we restricted the FDR threshold to less than 0.05 (−log10 of FDR more than 0.69) and set the log2 enrichment ratio as more than 2. These stringent criteria filtered out all terms except the GO biological processes “regulation of signal transduction by p53 class mediator” and “TOR signaling”. If we relaxed the threshold for log2 enrichment ratio to 1, then many other terms also met the requirement, including “mTOR signaling pathway”, “autophagy”, and “adipocytokine signaling pathway”, which, as mentioned above, have all been shown to be involved in gene regulation under hypoxia. This could suggest that the other pathways highlighted by this analysis, such as “AMPK signaling pathway”, “adipocytokine signaling pathway”, and “FoxO signaling pathway” have potential for further study. 

Nevertheless, this study does present some shortcomings that should be addressed in the future. Firstly, we focused on patterns of sexual differentiation only in villous cytotrophoblasts from first-trimester placentas, while previous research has revealed such patterns in different cell types, such as trophoblast epithelium and villous vessel endothelium from term placenta [51]. Secondly, we encountered the same problem as Soncin et al. [26], namely, that the collected samples did not cluster into distinct early and late groups (Figure 1c and Supplementary Figure S7a). This might be due to variations within groups or similarities between groups, which would have weakened the accuracy of the filtering process for key genes. Thirdly, because we aimed only to provide an overview of the changes in a specific period of pregnancy, we did not conduct any manipulative experiments to verify the role of selected key genes, although we did compare the individual expression patterns of certain critical genes (see Figure 6). Last but not least, we applied the TruSeq RNA Sample Prep Kit in our study instead of the TruSeq RNA Access Sample Prep Kit. The latter has a much lower amount of offtargeting than the former and enables the consolidation of library preparation for variant calling, fusion detection, and circular RNA identification, which plays a crucial role in development/cellular responses. Despite providing an overview of transcriptome of mRNA, the biased selection towards mRNA should be addressed as a drawback of the study.

## 4. Materials and Methods

### 4.1. Sample Collection and Ethics Statement

A total of 20 human placentas were collected from normal gravidas in the first trimester. To characterize the processes that accompany the increase in physiological oxygen tension from 8 to 14 GW, we investigated the early and late stages of this period, 8–10 GW and 12–14 GW, respectively. Equal numbers of female and male placentas were collected for each stage. We used the letter “E” to represent “Early”, “L” for “Late”, “F” for “Female”, and “M” for “Male”. The final sample pool contained 5 “EF” samples, 5 “EM” samples, 5 “LF” samples, and 5 “LM” samples. All placental tissues were obtained from patients who voluntarily and legally chose to terminate pregnancy during the first trimester at the Cochin Port-Royal (Paris, France). These biological samples were obtained following informed written consent from patients and approval from our local ethics committee (Comité de Protection des Personnes 909, 13 May 2015).

### 4.2. Cytotrophoblast Isolation and Culture

The first step was to isolate villous cytotrophoblasts from each sample. To do this, we purified the villous tissues by mincing them into pieces with forceps, removing the membrane on the surface, and rinsing them with Ca^2+^-, Mg^2+^-free Hanks’ balanced salt solution. The pieces were then digested in Hanks’ balanced salt solution with 4.2 mM MgSO_4_, 0.25% (wt/vol) trypsin powder (Difco, Rhône-Alpes, France), 5 IU of DNase I per mL, 25 mM HEPES, 100 IU/mL penicillin, and 100 μg/mL streptomycin (ThermoFisher, Waltham, MA, USA). Digestion was monitored by invert microscopy; the initial digested solution, which mainly consisted of red blood cells, was discarded and the subsequent digested solution, which mainly consisted of villous cytotrophoblasts, was retained for stratification. The digested solution was slowly transferred to a discontinuous Percoll gradient (5–70% in steps of 5%) in order to stratify the mixed cells and debris. The layer containing the cytotrophoblasts was retained and washed using Dulbecco’s modified Eagle’s medium (F12/DMEM), which contained 100 IU/mL penicillin, 100 mg/mL streptomycin, and 2 mM glutamine. Cells were counted using a TC20™ Automated Cell Counter (Biorad, Hercules, CA, USA). For RNASeq experiments, 1.5 × 10^6^ cells were transferred into a 1.5 mL Eppendorf tube. After centrifugation (a 10 s pulse at 14,000 rpm), the supernatants were discarded and the cell pellets were snap-frozen in liquid nitrogen and stored at −80 °C until total RNA extraction, PCR, and RNAseq analyses were conducted.

### 4.3. Fetal-Sex Determination by PCR

Fetal-sex determination was performed via PCR on stored cytotrophoblast cells, as described previously [52]. Genomic DNA (gDNA) was extracted using the 25 mM NaOH/9.2 mM EDTA buffer. The sex of the placenta was genetically determined by PCR according to the sex-linked chromosome genes *ZFX* (GenBank Acc.No. NG_021253, NM_003410) and *ZFY* (GenBank Acc.No. NG_008113, NM_003411). The primers were ChrX-Y_F 5′-ATTTGTTCTAAGTCGCCATATTCTCT-3′, ChrX_R 5′-GAACACACTACT-GAGCAAAATGTATA-3′, and ChrY_R 5′-CATCTTTACAAGCTTGTAGACACACT-3′. Reagents for PCR reactions included gDNA (100–300 ng) 10 µL 5×green GoTaq reaction buffer, 2 µL 10 mM dNTPs, 2.5 µL 10 µM ChrX-Y_F, 2.5 µL 10 µM ChrX_R, 2.5 µL 10 µM ChrY_R, 0.2 µL (5 u/µL) GoTaq DNA polymerase (Promega, Madison, WI, USA), and water to reach a total volume of 50 µL. Amplification was conducted in a Perkin Elmer Applied Biosystems GeneAmp PCR Thermal Cycler System 2700, with the following cycling parameters: initial denaturation at 94 °C for 10 min, 35 cycles of denaturation at 94 °C for 45 s, annealing at 50 °C for 45 s, and synthesis at 72 °C for 30 s, and an extension of 5 min at the end of the final cycle. Amplification products (10 µL) were directly analyzed on 2% agarose gel and evaluated under UV light. Primers ChrX-Y_F/ChrY_R were present only in male samples and primers ChrX-Y_F/ChrX_R were present in all tested samples. For validation, we also analyzed the expression of the sex-linked genes *XIST* and *DDX3Y* in the RNAseq dataset, following the procedures described in [53].

### 4.4. RNA-Seq Experiment and Data Processing

RNAseq analyses were performed as described in [54]. The RNeasy Micro Kit (Qiagen, Hilden, Germany) was used to extract total RNA from villous cytotrophoblasts isolated from placentas. DNAse was used to degrade genomic DNA, following the RNeasy Micro Kit protocol. RNA sequencing was performed by the Genom’IC lab facility of the Institut Cochin (Paris, France). RNA concentrations were quantified using a Nanodrop device (Thermo Fisher Scientific, Waltham, MA, USA) and the quality of the RNA was measured on an Agilent 2100 Bioanalyzer (Agilent Technologies, Palo Alto, CA, USA). A total of 800 ng RNA sample was used to construct each RNAseq library using the TruSeq Stranded mRNA kit (Illumina, San Diego, CA, USA). RNAseq libraries were quantified by RT-qPCR using the KAPA Library Quantification Kit for Illumina Libraries (KapaBiosystems, Wilmington, MA, USA) and corresponding profiles were evaluated using the DNA High Sensitivity LabChip kit on an Agilent Bioanalyzer. RNAseq libraries were sequenced on an Illumina Nextseq 500 instrument using 75 base-length reads and V2 chemistry, in paired-end mode. AOZAN software (ENS, Paris, France) was applied to demultiplex and characterize the raw data (based on FastQC module/version 0.11.5, Illumina, San Diego, CA, USA), and the obtained fastq sequence files were aligned using the STAR algorithm (version 2.5.2b, NY, USA). Raw reads were counted using Featurecount (version Rsubread 1.24.1, Melbourne, Australia) and processed as follows: (i) rows with reads equal to zero in more than 10 samples were excluded; (ii) all read counts were increased by 1 and log2-transformed; (iii) data were normalized using the DESeq2 package [55].

### 4.5. Clustering Analysis and Construction of Co-Expression Modules of Human Placental RNAseq Data

To detect outliers, sample clustering was performed based on Euclidean distance, principal component analysis (PCA), and t-distributed stochastic neighbor embedding (t-SNE). To identify differentially abundant genes, DESeq2 and WGCNA were applied separately and the results were then merged for a combined analysis. For DESeq2, gene expression was compared between the different groups, and the criteria for defining key genes were a fold-change of 2 and a *p*-value less than 0.05. For WGCNA, normalized RNAseq reads from all samples were submitted directly to the WGCNA package (version: 1.68) [56] in R (version: 4.0.1, R Core Team, Vienna, Austria) to evaluate correlations in gene expression. We initially assessed the optimal soft thresholding power value by using a range of power values from 1 to 20; the optimal value is the one for which the measurement of scale independence surpasses the threshold of 0.9. This optimal value was then used to reduce the background noise of the correlations in the adjacency matrix. The correlations of eigengenes from the adjacency matrix based on the default unsigned network were used to construct co-expression modules. The default minimum module size of 30 was used to increase the reliability of the results.

### 4.6. Analysis of Co-Expression Modules in Human Placental RNAseq Data

Each module—a set of topologically correlated genes—was represented by a different color. A clustering dendrogram was created based on the correlations between the genes of different modules and a module-trait heatmap was constructed based on the correlations between the module eigengenes and traits of interest (sex and age, i.e., the difference between the periods of 8–10 GW and 12–14 GW). For each module, we created scatterplots of the modules’ eigengenes in which the x-coordinate represented module membership (the correlation coefficient between a gene’s expression profile and the module eigengene) and the y-coordinate represented gene significance (GS; the correlation coefficient between the genes’ expression and the traits of interest). Only correlation coefficients higher than 0.6, with a *p*-value less than 0.05, were included for analyses of module membership.

### 4.7. Enrichment and Differential Abundance of Key Genes

Venn diagrams were created to depict the intersections between different datasets using the online tool Draw Venn diagram (http://bioinformatics.psb.ugent.be/webtools/Venn/). Enrichment in gene ontology (GO) terms and pathways was analyzed using the R package clusterProfiler [57] (version 3.9, synced to latest GO terms and pathways). The top 15 category terms for each group were assembled for comparison; those with a *p*-value lower than 0.05 were identified as being significantly enriched. WebGestalt was used to visualize the terms in volcano format and perform the network topology analysis [58]. Finally, to investigate the involvement of HIF (including both the alpha and beta subunits) in the biological processes under consideration, we retrieved all published or predicted targets of HIF-1α and HIF-1β from the literature and public databases using the R package *tftargets* (https://github.com/slowkow/tftargets). These were then assembled into a local database of HIF targets.

## 5. Conclusions

In conclusion, our results provide a broad perspective of the biological processes that are active in trophoblasts during the surge in physiological oxygen availability, specifically with regard to differences over time and between the sexes, which should open new avenues for future research and contribute to the discovery of possible drug-targeted genes or pathways in the human placenta.

## Data Availability

Our RNAseq dataset has been deposited in the Gene Expression Omnibus Public Repository (https://www.ncbi.nlm.nih.gov/geo/) under the accession number GSE163023 (https://www.ncbi.nlm.nih.gov/geo/query/acc.cgi?acc=GSE163023).

## Supplementary Materials

The following are available online at https://www.mdpi.com/1422-0067/22/6/2901/s1, The FastQC quality report for the dataset is in Figure S1, including information on phred scores, the average quality, the peaks and the normalization. Heatmaps of gene expression for the key genes identified by DESeq2 for the comparisons of different groups are shown in Figure S2a–f. The enriched terms of GO cellular components and GO molecular functions for the DESeq2 comparison are shown in Figure S3. From the WGCNA analysis, the expression of key genes in the modules is shown in separate heatmaps based on age and sex in Figure S4. The enriched terms of GO cellular components and GO molecular functions for the combined comparison are shown in Figure S5. Details of the evaluation of GO and KEGG pathway enrichment in the HIF targets are shown in Figure S6. A summary of dataset GSE100051 is shown in Figure S7, including the clustering of the collected samples, the expression of selected key genes, and a comparison of the terms. A detailed list of sources for the targets of HIF-1α and HIF-1β retrieved from the literature and public databases can be found in Table S1.

## References

[1] Soncin F., Natale D., Parast M.M. (2015). Signaling pathways in mouse and human trophoblast differentiation: A comparative review. Cell. Mol. Life Sci..

[2] James J.L., Carter A.M., Chamley L.W. (2012). Human placentation from nidation to 5 weeks of gestation. Part I: What do we know about formative placental development following implantation?. Placenta.

[3] Patel J., Landers K., Mortimer R.H., Richard K. (2010). Regulation of hypoxia inducible factors (HIF) in hypoxia and normoxia during placental development. Placenta.

[4] Jauniaux E., Watson A.L., Hempstock J., Bao Y.P., Skepper J.N., Burton G.J. (2000). Onset of maternal arterial blood flow and placental oxidative stress. A possible factor in human early pregnancy failure. Am. J. Pathol..

[5] Sibai B., Dekker G., Kupferminc M. (2005). Pre-eclampsia. Lancet.

[6] Goldman-Wohl D., Yagel S. (2002). Regulation of trophoblast invasion: From normal implantation to pre-eclampsia. Mol. Cell. Endocrinol..

[7] Ali K.Z. (1997). Stereological study of the effect of altitude on the trophoblast cell populations of human term placental villi. Placenta.

[8] Todros T., Sciarrone A., Piccoli E., Guiot C., Kaufmann P., Kingdom J. (1999). Umbilical Doppler waveforms and placental villous angiogenesis in pregnancies complicated by fetal growth restriction. Obstet. Gynecol..

[9] Schneider H. (2000). Placental oxygen consumption. Part II: In vitro studies—A review. Placenta.

[10] Carter A.M. (2000). Placental oxygen consumption. Part I: In vivo studies—A review. Placenta.

[11] Miller C.N., Dye J.A., Henriquez A.R., Stewart E.J., Lavrich K.S., Carswell G.K., Ren H., Freeborn D.L., Snow S.J., Schladweiler M.C. (2020). Ozone-induced fetal growth restriction in rats is associated with sexually dimorphic placental and fetal metabolic adaptation. Mol. Metab..

[12] Holland O., Dekker Nitert M., Gallo L.A., Vejzovic M., Fisher J.J., Perkins A.V. (2017). Review: Placental mitochondrial function and structure in gestational disorders. Placenta.

[13] Song H., Telugu B.P., Thompson L.P. (2019). Sexual dimorphism of mitochondrial function in the hypoxic guinea pig placenta. Biol. Reprod..

[14] Sferruzzi-Perri A.N., Camm E.J. (2016). The Programming Power of the Placenta. Front. Physiol..

[15] Burton G.J., Fowden A.L. (2015). The placenta: A multifaceted, transient organ. Philos. Trans. R. Soc. B Biol. Sci..

[16] Chang C.W., Wakeland A.K., Parast M.M. (2018). Trophoblast lineage specification, differentiation and their regulation by oxygen tension. J. Endocrinol..

[17] Mandl M., Depping R. (2014). Hypoxia-inducible aryl hydrocarbon receptor nuclear translocator (ARNT) (HIF-1beta): Is it a rare exception?. Mol. Med..

[18] Lee J.W., Bae S.H., Jeong J.W., Kim S.H., Kim K.W. (2004). Hypoxia-inducible factor (HIF-1)alpha: Its protein stability and biological functions. Exp. Mol. Med..

[19] Adelman D.M., Gertsenstein M., Nagy A., Simon M.C., Maltepe E. (2000). Placental cell fates are regulated in vivo by HIF-mediated hypoxia responses. Genes Dev..

[20] Muralimanoharan S., Maloyan A., Myatt L. (2013). Evidence of sexual dimorphism in the placental function with severe preeclampsia. Placenta.

[21] Huppertz B. (2008). Placental origins of preeclampsia: Challenging the current hypothesis. Hypertension.

[22] Roberts J.M., Cooper D.W. (2001). Pathogenesis and genetics of pre-eclampsia. Lancet.

[23] Braun A.E., Muench K.L., Robinson B.G., Wang A., Palmer T.D., Winn V.D. (2021). Examining Sex Differences in the Human Placental Transcriptome During the First Fetal Androgen Peak. Reprod. Sci..

[24] Gonzalez T.L., Sun T., Koeppel A.F., Lee B., Wang E.T., Farber C.R., Rich S.S., Sundheimer L.W., Buttle R.A., Chen Y.I. (2018). Sex differences in the late first trimester human placenta transcriptome. Biol. Sex Differ..

[25] Sitras V., Fenton C., Paulssen R., Vartun A., Acharya G. (2012). Differences in gene expression between first and third trimester human placenta: A microarray study. PLoS ONE.

[26] Soncin F., Khater M., To C., Pizzo D., Farah O., Wakeland A., Arul Nambi Rajan K., Nelson K.K., Chang C.W., Moretto-Zita M. (2018). Comparative analysis of mouse and human placentae across gestation reveals species-specific regulators of placental development. Development.

[27] Sun T., Gonzalez T.L., Deng N., DiPentino R., Clark E.L., Lee B., Tang J., Wang Y., Stripp B.R., Yao C. (2020). Sexually Dimorphic Crosstalk at the Maternal-Fetal Interface. J. Clin. Endocrinol. Metab..

[28] Brew O., Sullivan M.H.F. (2017). Oxygen and tissue culture affect placental gene expression. Placenta.

[29] Cross C.E., Tolba M.F., Rondelli C.M., Xu M., Abdel-Rahman S.Z. (2015). Oxidative Stress Alters miRNA and Gene Expression Profiles in Villous First Trimester Trophoblasts. BioMed Res. Int..

[30] Treissman J., Yuan V., Baltayeva J., Le H.T., Castellana B., Robinson W.P., Beristain A.G. (2020). Low oxygen enhances trophoblast column growth by potentiating differentiation of the extravillous lineage and promoting LOX activity. Development.

[31] Liu F., Zhu W., Shoaito H., Chissey A., Degrelle S.A., Fournier T. (2020). Mining of combined human placental gene expression data across pregnancy, applied to PPAR signaling pathway. Placenta.

[32] Qi L., Liu B., Chen X., Liu Q., Li W., Lv B., Xu X., Wang L., Zeng Q., Xue J. (2020). Single-Cell Transcriptomic Analysis Reveals Mitochondrial Dynamics in Oocytes of Patients With Polycystic Ovary Syndrome. Front. Genet..

[33] Wang Z., Tang W., Yuan J., Qiang B., Han W., Peng X. (2020). Integrated Analysis of RNA-Binding Proteins in Glioma. Cancers.

[34] Koh M.J., Shin D.H., Lee S.J., Hwang C.S., Lee H.J., Kim A., Park W.Y., Lee J.H., Choi K.U., Kim J.Y. (2020). Gastric-type gene expression and phenotype in non-terminal respiratory unit type adenocarcinoma of the lung with invasive mucinous adenocarcinoma morphology. Histopathology.

[35] Broderick S.R., Wijeratne S., Wijeratn A.J., Chapin L.J., Meulia T., Jones M.L. (2014). RNA-sequencing reveals early, dynamic transcriptome changes in the corollas of pollinated petunias. BMC Plant Biol..

[36] Zhang X., Fu L.J., Liu X.Q., Hu Z.Y., Jiang Y., Gao R.F., Feng Q., Lan X., Geng Y.Q., Chen X.M. (2016). nm23 regulates decidualization through the PI3K-Akt-mTOR signaling pathways in mice and humans. Hum. Reprod..

[37] Saha B., Ganguly A., Home P., Bhattacharya B., Ray S., Ghosh A., Rumi M.A.K., Marsh C., French V.A., Gunewardena S. (2020). TEAD4 ensures postimplantation development by promoting trophoblast self-renewal: An implication in early human pregnancy loss. Proc. Natl. Acad. Sci. USA.

[38] Chang C.W., Cheong M.L., Chang G.D., Tsai M.S., Chen H. (2013). Involvement of Epac1/Rap1/CaMKI/HDAC5 signaling cascade in the regulation of placental cell fusion. Mol. Hum. Reprod..

[39] Gerbaud P., Tasken K., Pidoux G. (2015). Spatiotemporal regulation of cAMP signaling controls the human trophoblast fusion. Front. Pharmacol..

[40] Haider S., Meinhardt G., Velicky P., Otti G.R., Whitley G., Fiala C., Pollheimer J., Knofler M. (2014). Notch signaling plays a critical role in motility and differentiation of human first-trimester cytotrophoblasts. Endocrinology.

[41] Ietta F., Bechi N., Romagnoli R., Bhattacharjee J., Realacci M., Di Vito M., Ferretti C., Paulesu L. (2010). 17β-Estradiol modulates the macrophage migration inhibitory factor secretory pathway by regulating ABCA1 expression in human first-trimester placenta. Am. J. Physiol. Endocrinol. Metab..

[42] Kho A.T., Chhabra D., Sharma S., Qiu W., Carey V.J., Gaedigk R., Vyhlidal C.A., Leeder J.S., Tantisira K.G., Weiss S.T. (2016). Age, Sexual Dimorphism, and Disease Associations in the Developing Human Fetal Lung Transcriptome. Am. J. Respir. Cell. Mol. Biol..

[43] Baczyk D., Audette M.C., Coyaud E., Raught B., Kingdom J.C. (2018). Spatiotemporal distribution of small ubiquitin-like modifiers during human placental development and in response to oxidative and inflammatory stress. J. Physiol..

[44] Bebington C., Doherty F.J., Fleming S.D. (1999). Ubiquitin cross-reactive protein gene expression is increased in decidualized endometrial stromal cells at the initiation of pregnancy. Mol. Hum. Reprod..

[45] Anton L., Brown A.G., Bartolomei M.S., Elovitz M.A. (2014). Differential methylation of genes associated with cell adhesion in preeclamptic placentas. PLoS ONE.

[46] Mohammad N., Yaqinuddin A., Kakal F., Sheikh L., Qureshi R., Somani M. (2013). Frequent hypomethylation of PTGS2 gene promoter in human term placenta. Ital. J. Anat. Embryol..

[47] Ke Q., Costa M. (2006). Hypoxia-inducible factor-1 (HIF-1). Mol. Pharmacol..

[48] Yamanaka-Tatematsu M., Nakashima A., Fujita N., Shima T., Yoshimori T., Saito S. (2013). Autophagy induced by HIF1alpha overexpression supports trophoblast invasion by supplying cellular energy. PLoS ONE.

[49] Gauster M., Maninger S., Siwetz M., Deutsch A., El-Heliebi A., Kolb-Lenz D., Hiden U., Desoye G., Herse F., Prokesch A. (2018). Downregulation of p53 drives autophagy during human trophoblast differentiation. Cell. Mol. Life Sci..

[50] Perkins J., St John J., Ahmed A. (2002). Modulation of trophoblast cell death by oxygen and EGF. Mol. Med..

[51] Cvitic S., Longtine M.S., Hackl H., Wagner K., Nelson M.D., Desoye G., Hiden U. (2013). The human placental sexome differs between trophoblast epithelium and villous vessel endothelium. PLoS ONE.

[52] Degrelle S.A., Fournier T. (2018). Fetal-sex determination of human placental tissues. Placenta.

[53] Hoch D., Novakovic B., Cvitic S., Saffery R., Desoye G., Majali-Martinez A. (2020). Sex matters: XIST and DDX3Y gene expression as a tool to determine fetal sex in human first trimester placenta. Placenta.

[54] Rachdi L., Maugein A., Pechberty S., Armanet M., Hamroune J., Ravassard P., Marullo S., Albagli O., Scharfmann R. (2020). Regulated expression and function of the GABAB receptor in human pancreatic beta cell line and islets. Sci. Rep..

[55] Love M.I., Huber W., Anders S. (2014). Moderated estimation of fold change and dispersion for RNA-seq data with DESeq2. Genome Biol..

[56] Langfelder P., Horvath S. (2008). WGCNA: An R package for weighted correlation network analysis. BMC Bioinform..

[57] Yu G., Wang L.G., Han Y., He Q.Y. (2012). clusterProfiler: An R package for comparing biological themes among gene clusters. OMICS.

[58] Liao Y., Wang J., Jaehnig E.J., Shi Z., Zhang B. (2019). WebGestalt 2019: Gene set analysis toolkit with revamped UIs and APIs. Nucleic Acids Res..

