# Supplementary material for: Age and Sex-Related Changes in Human First-Trimester Placenta Transcriptome and Insights into Adaptative Responses to Increased Oxygen
Source: Int J Mol Sci. 2021 Mar 12;22(6):2901. doi: 10.3390/ijms22062901 (PMC8001632; doi:10.3390/ijms22062901)
Supplement: Supplementary file 1 [file Figure S2.pdf › Figure S2.pdf]

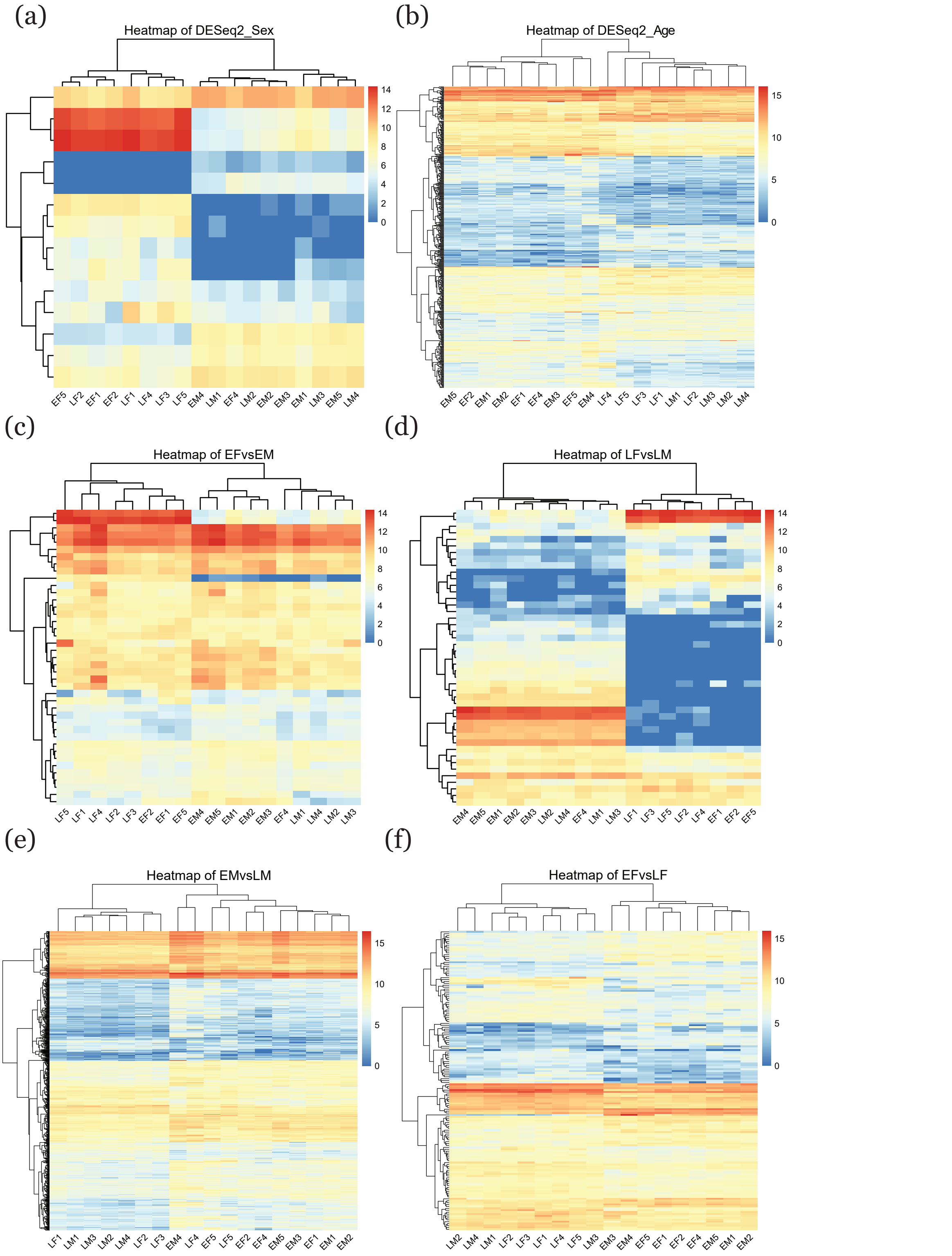

Figure S2. The heatmaps of gene expression for the key genes identified using DESeq2 under the comparison of different groups, including Female vesus Male (a), Early versus Late (b), EF versus EM (c), LF versus LM (d), EM versus LM (e), and EF versus LF (f). Alphabet “E” represents the abbreviation of “Early”, “L” for “Late”, “F” for “Female” and “M” for “Male” in groups.
