# Supplementary material for: Age and Sex-Related Changes in Human First-Trimester Placenta Transcriptome and Insights into Adaptative Responses to Increased Oxygen
Source: Int J Mol Sci. 2021 Mar 12;22(6):2901. doi: 10.3390/ijms22062901 (PMC8001632; doi:10.3390/ijms22062901)
Supplement: Supplementary file 1 [file ijms-22-02901-s001.zip › Figure S3.pdf]

(a)

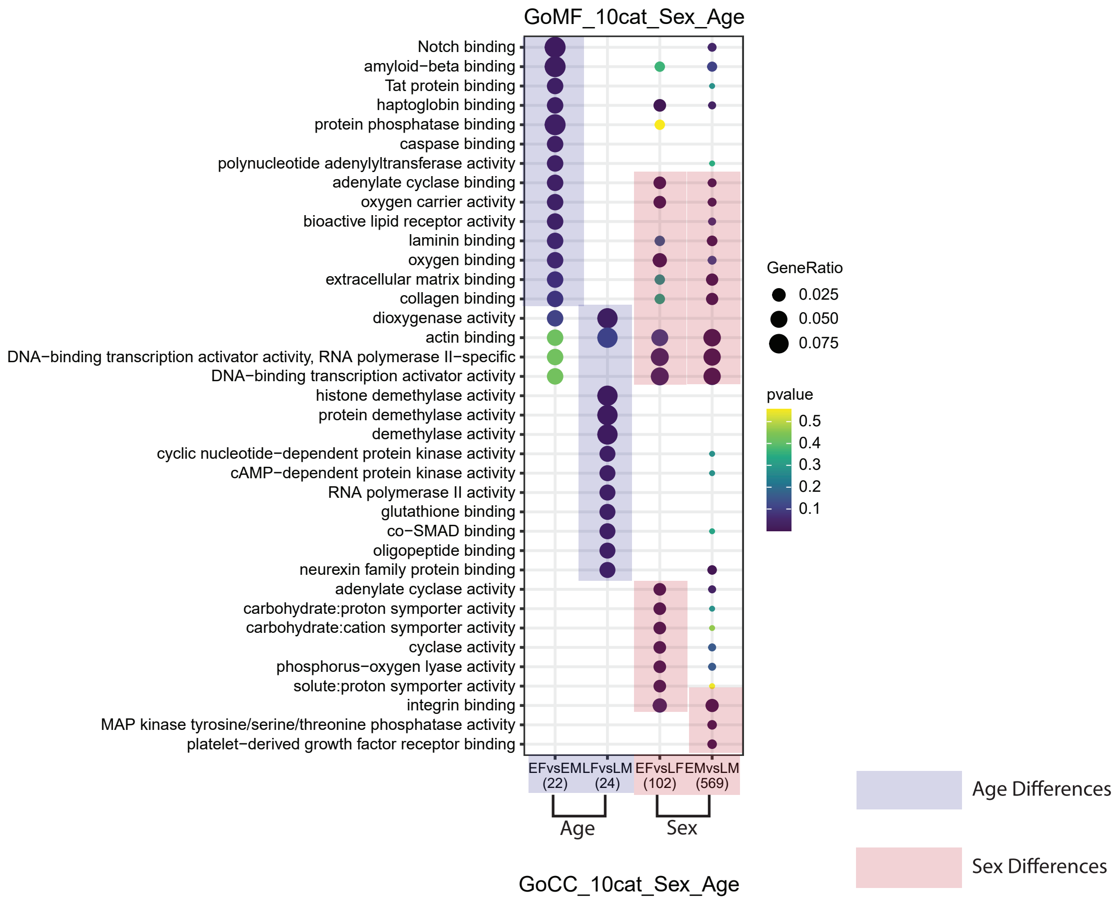

(b)

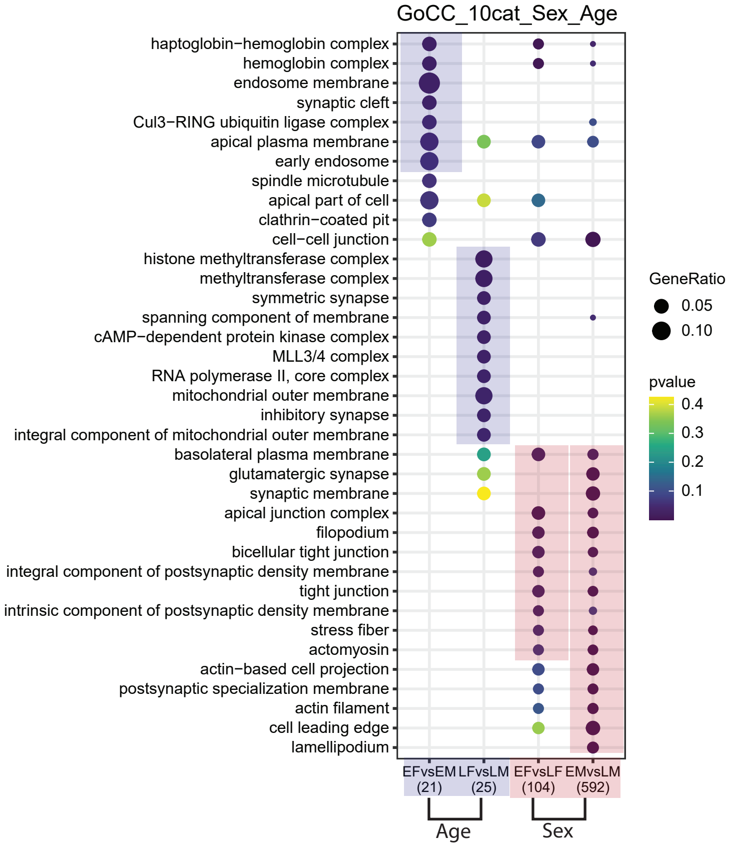

Figure S3. The enriched terms of GO cellular component (a) and GO molecular function (b) for the DESeq2 method's comparison. Weighted points filled with plum represent the age difference and coral represent the sexual difference. Alphabet "E" represents the abbreviation of "Early", "L" for "Late", "F" for "Female" and "M" for
