# Supplementary material for: Age and Sex-Related Changes in Human First-Trimester Placenta Transcriptome and Insights into Adaptative Responses to Increased Oxygen
Source: Int J Mol Sci. 2021 Mar 12;22(6):2901. doi: 10.3390/ijms22062901 (PMC8001632; doi:10.3390/ijms22062901)
Supplement: Supplementary file 1 [file ijms-22-02901-s001.zip › Figure S4.pdf]

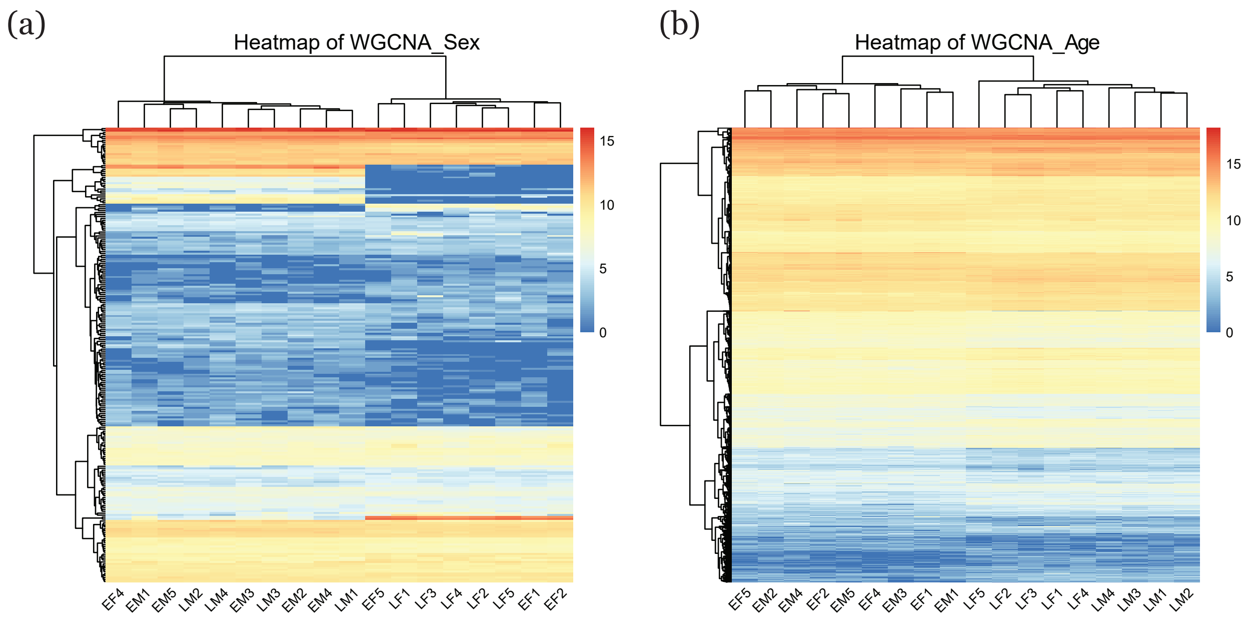

Figure S4. The expressions of key genes in the modules are shown in heatmap separately using WGCNA based on the traits of age (a) and sexes (b). WGCNA: weighted gene co-expression network analysis.
