# Supplementary material for: Age and Sex-Related Changes in Human First-Trimester Placenta Transcriptome and Insights into Adaptative Responses to Increased Oxygen
Source: Int J Mol Sci. 2021 Mar 12;22(6):2901. doi: 10.3390/ijms22062901 (PMC8001632; doi:10.3390/ijms22062901)
Supplement: Supplementary file 1 [file ijms-22-02901-s001.zip › Figure S5.pdf]

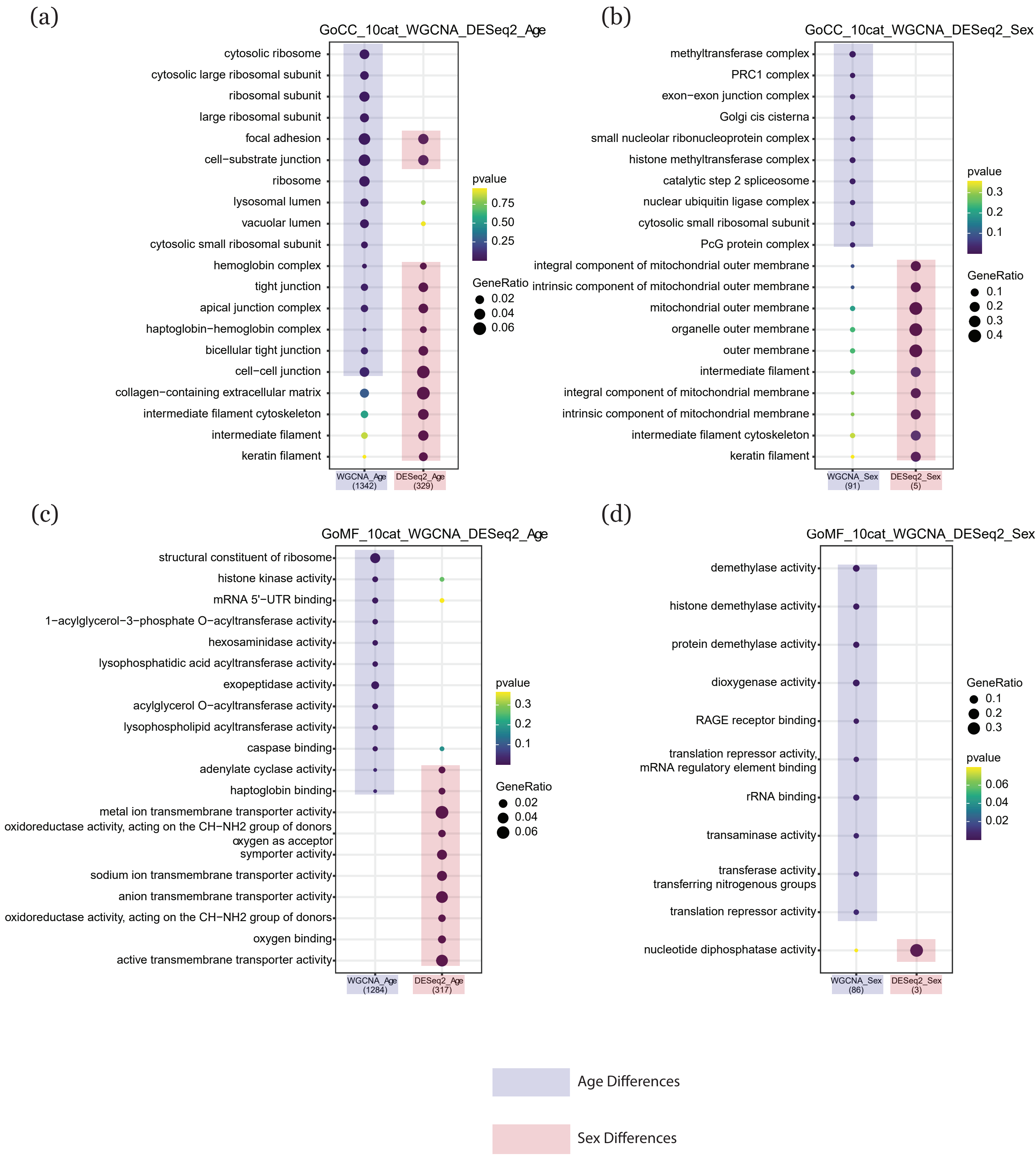

Figure S5. Comparison of enriched GO cellular component (CC) and GO molecular function (MF) based on genes selected by DESeq2 and WGCNA methods separately. (a&c) Enrichment and comparison of terms under age difference. (b&d) Enrichment and comparison of terms nder sexual difference. Weighted points filled with plum represent the age difference and coral represent the sexual difference. GO: gene
