# Supplementary material for: Age and Sex-Related Changes in Human First-Trimester Placenta Transcriptome and Insights into Adaptative Responses to Increased Oxygen
Source: Int J Mol Sci. 2021 Mar 12;22(6):2901. doi: 10.3390/ijms22062901 (PMC8001632; doi:10.3390/ijms22062901)
Supplement: Supplementary file 1 [file ijms-22-02901-s001.zip › Figure S6.pdf]

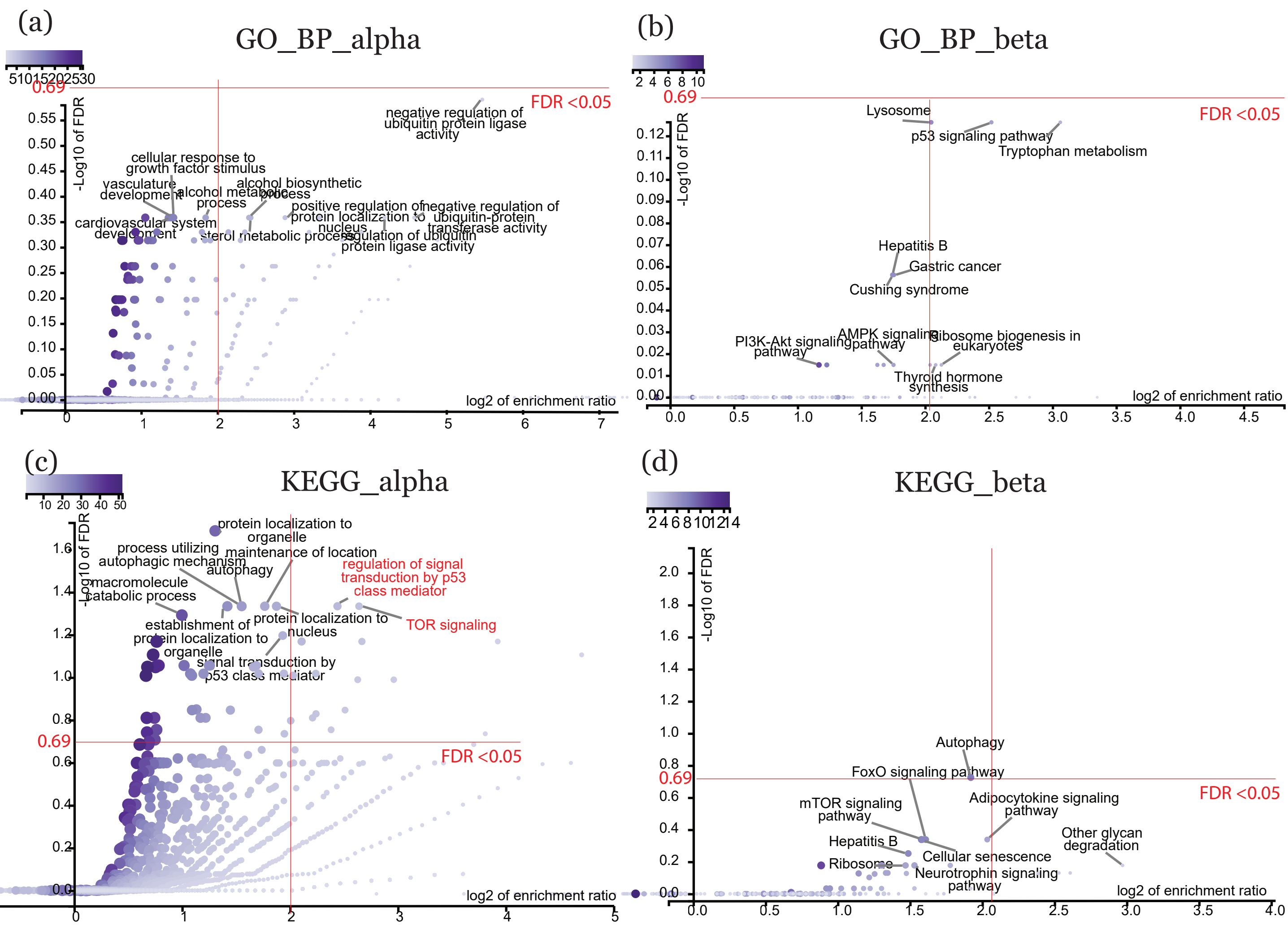

Figure S6. Details of evaluation for the terms of GO and KEGG pathways for the HIF targets. (a) Scatterplot of GO_BP_alpha. (b) Scatterplot of GO_BP_beta. (c) Scatterplot of KEGG_alpha. (d) Scatterplot of KEGG_beta. Y coordinate represents -log10 of FDR and x coordinate represents log2 enrichment ratio. GO: gene ontology; BP: biological process; HIF: hypoxia-inducible factor; KEGG: Kyoto encyclopedia of genes and genomes.
