# Supplementary material for: Age and Sex-Related Changes in Human First-Trimester Placenta Transcriptome and Insights into Adaptative Responses to Increased Oxygen
Source: Int J Mol Sci. 2021 Mar 12;22(6):2901. doi: 10.3390/ijms22062901 (PMC8001632; doi:10.3390/ijms22062901)
Supplement: Supplementary file 1 [file ijms-22-02901-s001.zip › Figure S7.pdf]

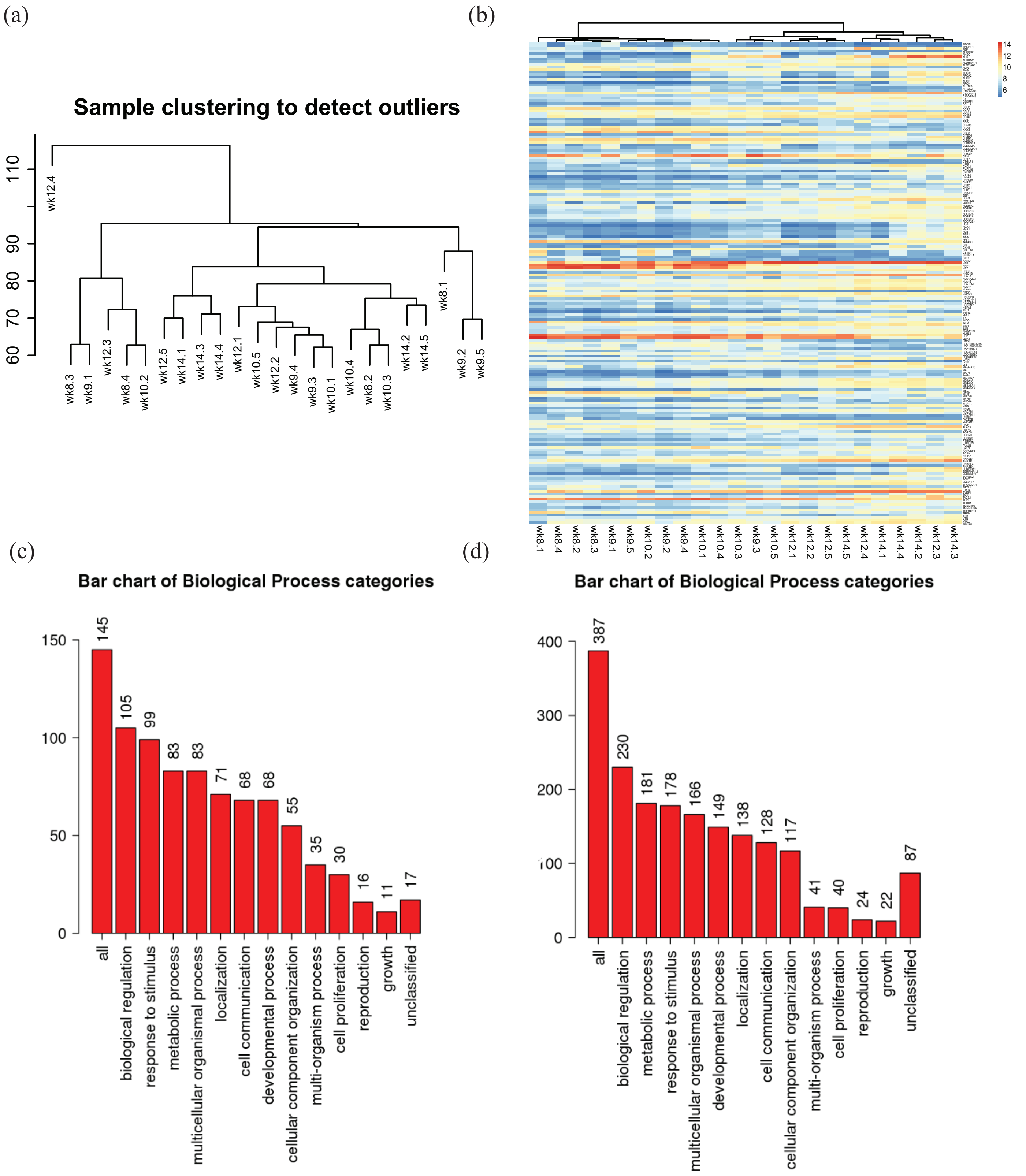

Figure S7. Summary of dataset GSE100051. The clustering of the collected samples from GSE100051 (a). The expressions of the selected key genes. (b) The comparison of the terms from GSE100051 and our results using the DESeq2 analysis (c&d).
