# Supplementary material for: Age and Sex-Related Changes in Human First-Trimester Placenta Transcriptome and Insights into Adaptative Responses to Increased Oxygen
Source: Int J Mol Sci. 2021 Mar 12;22(6):2901. doi: 10.3390/ijms22062901 (PMC8001632; doi:10.3390/ijms22062901)
Supplement: Supplementary file 1 [file ijms-22-02901-s001.zip › Table S1.pdf]

Supplementary Materials

Age and Sex-Related Changes in Human First-Trimester Placenta Transcriptome and Insights into Adaptative Responses to Oxygen Rising

Fulin Liu, Christelle Simasotchi, Françoise Vibert, Wencan Zhu, Sophie Gil, Séverine A. Degrelle and Thierry Fournier

Table S1. Datasets for extracting hypoxia-inducible factors (HIF) targets.

Dataset	Gene Identifier	Number of TF	Number of TF Targets	Reference
TRED	ENTREZ	133	7066	Jiang et al. [1]
ITFP	HGNC Symbol/Alias	1974	67154	Zheng et al. [2]
ENCODE	ENTREZ	157	20428	Encode [3]
Neph2012	HGNC Symbol/Alias	536	16484	Neph et al.[4]
TRRUST	HGNC Symbol/Alias	748	8215	Han et al. [5]
Marbach2016	HGNC Symbol/Alias	643	1305782	Marbach et al. [6]

References

1. Jiang, C.; Xuan, Z.; Zhao, F.; Zhang, M.Q. TRED: a transcriptional regulatory element database, new entries and other development. *Nucleic Acids Res.* **2007**, *35* (Database issue), D137–D140.
2. Zheng, G.; Tu, K.; Yang, Q.; Xiong, Y.; Wei, C.; Xie, L.; Zhu, Y.; Li, Y. ITFP: an integrated platform of mammalian transcription factors. *Bioinformatics* **2008**, *24*, 2416–2417.
3. Consortium, E.P. An integrated encyclopedia of DNA elements in the human genome, *Nature* **2012**, *489*, 57–74.
4. Neph, S.; Stergachis, A.B.; Reynolds, A.; Sandstrom, R.; Borenstein, E.; Stamatoyannopoulos JA. Circuitry and dynamics of human transcription factor regulatory networks. *Cell* **2012**, *150*, 1274–1286.
5. Han, H.; Shim, H.; Shin, D.; Shim, J.E.; Ko, Y.; Shin, J.; Kim, H.; Cho, A.; Kim, E.; Lee, T. et al. TRRUST: a reference database of human transcriptional regulatory interactions. *Sci. Rep.* **2015**, *5*, 11432.
6. Marbach, D.; Lamparter, D.; Quon, G.; Kellis, M.; Kutalik, Z.; Bergmann, S. Tissue-specific regulatory circuits reveal variable modular perturbations across complex diseases. *Nat. Methods* **2016**, *13*, 366–370.
