# Supplementary material for: Age and Sex-Related Changes in Human First-Trimester Placenta Transcriptome and Insights into Adaptative Responses to Increased Oxygen
Source: Int J Mol Sci. 2021 Mar 12;22(6):2901. doi: 10.3390/ijms22062901 (PMC8001632; doi:10.3390/ijms22062901)
Supplement: Supplementary file 1 [file ijms-22-02901-s001.zip › Figure S1.pdf]

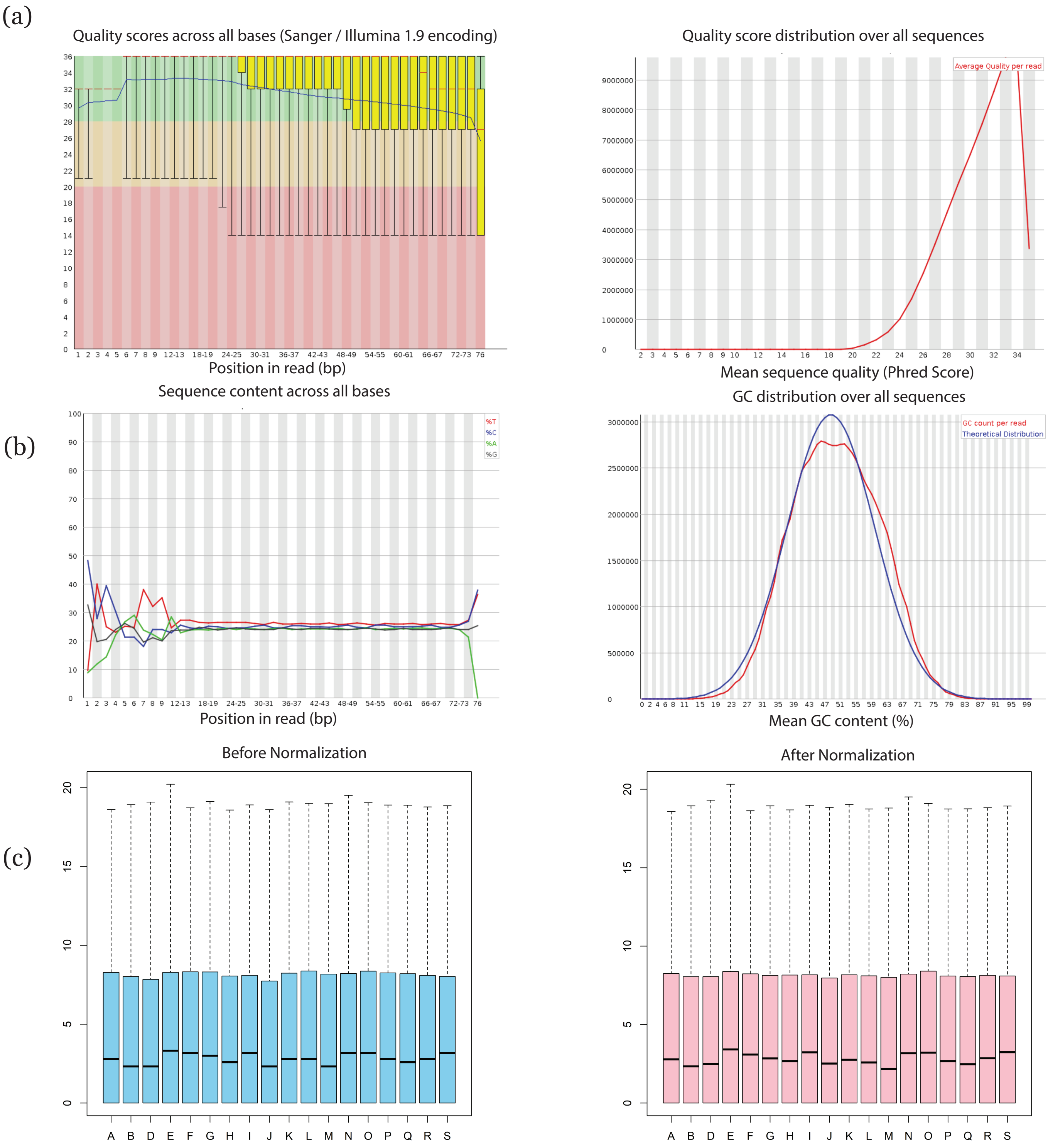

Figure S1. Quality report of raw data. (a) Average quality of sequencing for each position within the reads and the distribution according to their average quality. (b) Representation of the average proportion of each base for all reads and distribution of readings according to their composition in GC. (c) Normalization of raw data: before and after.
